# Sputtered LiCoO_2_ Cathode Materials for All-Solid-State Thin-Film Lithium Microbatteries

**DOI:** 10.3390/ma12172687

**Published:** 2019-08-22

**Authors:** Christian M. Julien, Alain Mauger, Obili M. Hussain

**Affiliations:** 1 Institut de Minéralogie, de Physique des Matériaux et de Cosmochimie (IMPMC), CNRS UMR 7590, Campus Pierre et Marie Curie, Sorbonne Université, 4 place Jussieu, 75005 Paris, France; 2 Thin Films Laboratory, Department of Physics, Sri Venkateswara University, Tirupati 517502, India

**Keywords:** thin films, sputtering technique, lithium cobaltate, cathode material, lithium microbattery

## Abstract

This review article presents the literature survey on radio frequency (RF)-magnetron sputtered LiCoO_2_ thin films used as cathode materials in all-solid-state rechargeable lithium microbatteries. As the process parameters lead to a variety of texture and preferential orientation, the influence of the sputtering conditions on the deposition of LiCoO_2_ thin films are considered. The electrochemical performance is examined as a function of composition of the sputter Ar/O_2_ gas mixture, gas flow rate, pressure, nature of substrate, substrate temperature, deposition rate, and annealing temperature. The state-of-the-art of lithium microbatteries fabricated by the rf-sputtering method is also reported.

## 1. Introduction

Rechargeable thin-film lithium microbatteries (LMBs) have been developed to power wearable electronic microdevices as a noise-free power source [1]. All-solid-state LMBs have attracted more and more interest due to safety and chemical stability issues of the solid electrolyte. The first microbattery made at Oak Ridge National Labs (ORNL) was integrated to complementary metal oxide semiconductor (CMOS) chips as a memory for the basic input/output system (BIOS) of computers [2,3,4]. Currently, there is an increasing demand for thin-film microbatteries. While the global Li-ion battery market is expected to surpass $77 billion by 2024, the thin-film batteries market is forecasted to reach $1.72 billion [5]. Since the invention by the ORNL group, numerous studies have been devoted to the design and optimization of the best positive electrode material, which is the limiting element of a microbattery (i.e., the capacity delivered by the power source is governed by the material). In particular, LiCoO_2_ (LCO), which was identified as a promising candidate cathode material for Li-ion batteries (LIBs) [6], yields a practical specific capacity of 135 mAh g^−1^ and it shows a fast charge-discharge reaction in the potential range from ~3.8 V (fully lithiated state) to ~4.2 V vs. Li^+^/Li (charge state at Li_0.5_CoO_2_) [7].

Among the various physical deposition techniques, radio-frequency (RF) magnetron sputtering is a powerful method to prepare oxide thin-films by controlling their crystalline structure and surface roughness with a composition similar to that of the target material. Features that make rf-magnetron sputtering a suitable technique for all-solid-state batteries can be summarized as follows. This deposition method can operate in a reactive atmosphere at low pressure (0.1 to 10 Pa). Using a high frequency of 13.56 MHz to adequately ionize the gas, there are reduced arcing and charge-up effects. Finally, due to the highly accelerated particles arriving on the substrate (10 to 40 eV), the rf-sputtered coatings have superior adhesion compared with other techniques. In addition, the chamber can include the multiple targets needed for successive deposits during the manufacture of a microbattery.

Various intercalation compounds such as thin-film cathodes for LMBs have been fabricated by rf-magnetron sputtering, i.e., MoO_3_, V_2_O_5_, LiMn_2_O_4_, LiCoO_2_, LifePO_4_, WO_3_, etc. [8,9,10,11]. Actually, sputtered LiCoO_2_ films have become the most popular electrodes for thin-film microbatteries. The choice of this material comes from several reasons. (i) It has demonstrated high electrochemical performance due to its lamellar structure favorable for Li^+^ ion pathway. (ii) It is a ternary compound whose synthesis is more easily controllable than other compound performers like LiFePO_4_ (LFP) and LiNi_x_Mn_y_Co_1-x-y_O_2_ (NMC). (iii) It has a higher voltage (>4 V vs. Li^+^/Li) and delivers higher energy density than LiFePO_4_ olivine or LiMn2O_4_ spinel. (iv) The growth with preferential orientation for fast kinetics is relatively easy.

**Figure 1:**
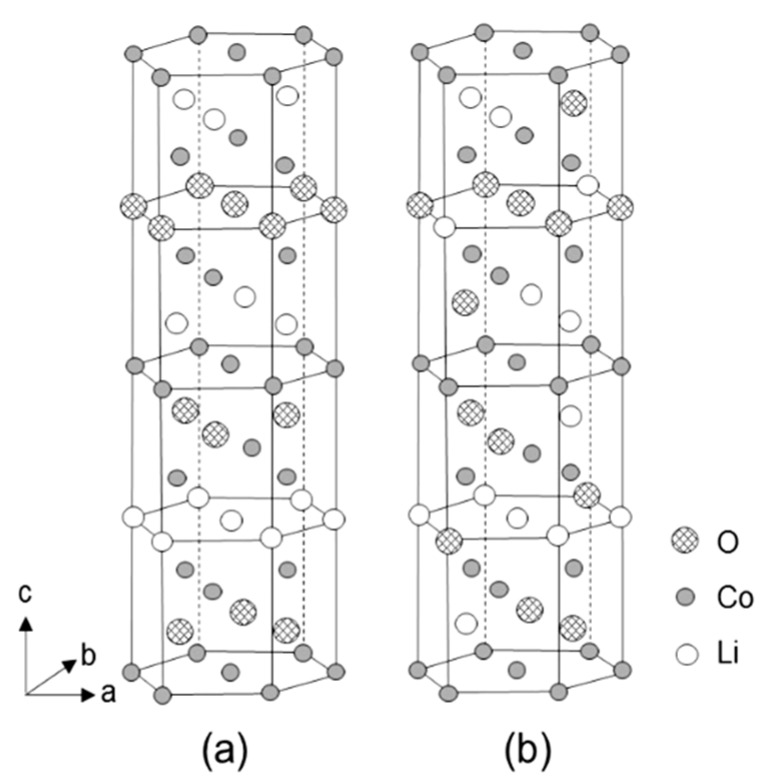
View of the structure of LiCoO_2_. (**a**) The high-temperature (HT) phase, rhombohedral structure (space group *R*-3*m*). (**b**) The low-temperature (LT) phase, cubic structure (space group *Fd*3*m*). Reproduced with permission from [14]. Copyright 1995 Elsevier.

LiCoO_2_ (LCO) crystallizes in the rhombohedral structure (space group *R*-3*m*) with atoms in the following Wykoff positions: Co in 3*a* (0,0,0), Li in 3*b* (0,0,½) and O in 6*c* sites (0,0,¼). Li and Co atoms are alternately located on octahedral sites between adjacent close-packed (*ccp*) planes of oxygen. The unit cell of the LCO structures is presented in Figure 1. The ordered structure is known as HT-LiCoO_2_ due to its formation at a temperature higher than 600 °C (Figure 1a), while the low-temperature modification (LT-LiCoO_2_) is slightly disordered with a cubic structure (space group *Fd*3*m*; Figure 1b). The existence of the HT-LiCoO_2_ structure is generally confirmed by a distinct splitting of the (110)/(108) doublet of the XRD diffraction lines. Note in the ideal *ccp* lattice the *c*/*a* ratio is 2√6 (4.899). For the well-crystallized HT-LiCoO_2_ phase synthesized by the sol-gel via malic acid assisted method, the c/a ratio is reported to be 4.987 [12]. Magnetic properties indicated that LiCoO_2_ consists of Co^3+^ (t_2g_^6^e_g_^0^) ions in the low-spin state [13].

**Figure 2:**
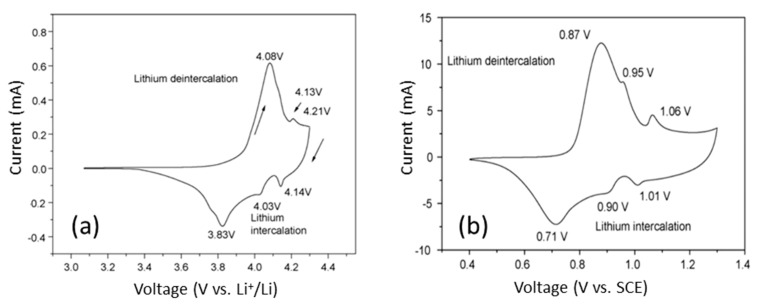
Cyclic voltammograms of Li//LiCoO_2_ obtained (**a**) in organic (aprotic) electrolyte at a scan rate of 0.1 mV s^−1^ and (**b**) in saturated Li_2_SO_4_ aqueous electrolyte at scan rate of 0.6 mV s^−1^. Reproduced with permission from [18]. Copyright 2009 Elsevier.

The investigation of electrochemical behavior of Li_x_CoO_2_ by the cyclic voltammetry or galvanostatic method in both non-aqueous (aprotic) and aqueous electrolytes demonstrated the phase changes in the potential window 3.0–4.3 V due to the (de)intercalation reactions in the composition range 1.0 ≥ *x* ≥ 0.5 [15,16]. As shown in Figure 2, a typical voltammogram of Li_x_CoO_2_ in organic electrolyte exhibits three anodic/cathodic couples. The broad peaks at 4.08/3.83 V are associated with the two-phase domain (0.75 ≥ *x* ≥ 0.95) and the two pairs of redox peaks at 4.13/4.03 and 4.21/4.14 V are related to the interlayer Li vacancy in-plane ordering [17]. LiCoO_2_ in a saturated Li_2_SO_4_ aqueous solution demonstrated similar electrochemical features [18].

Well-crystallized LCO films exhibit a clear discharge voltage plateau at ca. 4 V vs. Li^+^/Li, but the discharge profile appears sloping for amorphous-like films. Most of the commercial all solid-state thin-film lithium microbatteries constructed with a LCO cathode, a fast Li^+^-ion conductor as the solid electrolyte, and a metallic Li anode, achieve initial specific capacities of ~60 mAh cm^−2^ µm^−1^ on discharge, which is close to the theoretical value ~68.9 µAh cm^−2^ µm^−1^ or 248 mC cm^−2^ µm^−1^ (i.e., corresponding to gravimetric capacity of 137 mAh g^−1^ for a density of 5.06 g cm^−3^ and Li uptake *x* = 0.5 in Li_x_CoO_2_) [19].

Here, we report the structural and electrochemical properties of LiCoO_2_ thin films prepared by the rf-sputtering method, for which relationships are established between texture, experimental conditions, and performance as the cathode thin films. Applications of LCO films in lithium microbatteries are also examined. This paper is organized as follows: The all-solid-state lithium thin film batteries fabricated by the rf-sputtering technique are introduced in Section 2; the optimization of the growth of LiCoO_2_ thin films is presented in Section 3; a detailed examination of the influence of the sputtered conditions on the texture is given in Section 4 with an emphasis of the growth-parameter/microstructure relationship; the following thereof Section 5 reports the electrochemical properties (charge-discharge profiles and Li^+^ ions kinetics) and a brief overview of the properties of doped LiCoO_2_ thin films.

## 2. All Solid-State Lithium Microbatteries

**Figure 3:**
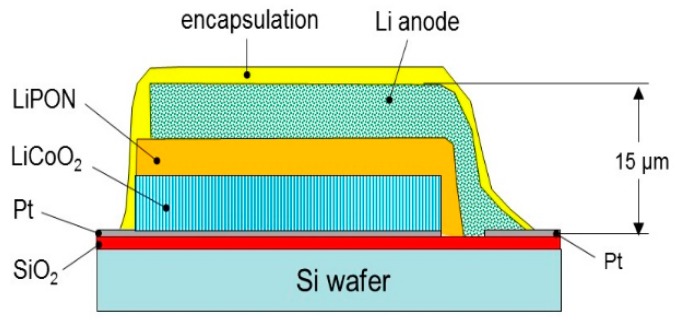
Schematic cross-section illustrating the layout of an all-solid-state thin-film battery.

Today, the all-solid-state lithium batteries using solid electrolytes are considered to be the new generation of rechargeable batteries [20], but advances in this kind of power sources are constant since the 1990s. A typical all-solid-state microbattery (SSMB) is constituted by three main active materials sequentially deposited as thin layers on a substrate: A ~1-µm thick metallic lithium as the negative electrode (anode), a ~2-µm thick solid-state inorganic electrolyte (SSE) as the separator, and a ~2–3-µm thick intercalation compound (IC) as the positive electrode (cathode). Alternative thin film anodes have been used such as SiSn_0.87_O_1.20_N_1.72_ (SiTON), Sn_3_N_4_, Zn_3_N_2_, or Cu films [21]. The total thickness of SSMBs is of the order of ~10–15 µm including current collector and encapsulation (Figure 3). Table 1 lists the characteristics of all-solid-state lithium microbatteries with LCO cathode fabricated by the rf-sputtering technique.

With respect to the requested low internal resistance, the challenges in a thin-film lithium cell arise from the low ionic conductivity of the SSE and the quality of the electrode-SSE interfaces. The most popular technique for the preparation of a cathode thin-film is radio-frequency magnetron sputtering (abbreviation RFS hereafter). The first SSMBs were developed using TiS_2_, V_2_O_5_, and LiMn_2_O_4_ as ICs [2] and the fast-ion conductor lithium phosphorous oxynitride (LiPON) as a solid electrolyte. This material with a typical composition of Li_3.3_PO_3.8_N_0.22_ exhibits an ionic conductivity of 2 µS cm^−1^ at 25 °C, and it is electrochemically stable up to 5.5 V vs. Li^+^/Li at room temperature [31]. The formation of additional cross-linking between PO_4_ groups and the low electrostatic energy of P–N bonds are the origin for high ionic conductivity [32]. Thin film of LiPON is easily fabricated by rf-magnetron sputtering of a Li_3_PO_4_ target in N_2_ atmosphere. Several groups have reported improved ionic transport for film with higher N content. Table 2 summarizes the N/P ratio dependence of the electrical properties of LiPON films prepared by RF-sputtering.

**Figure 4:**
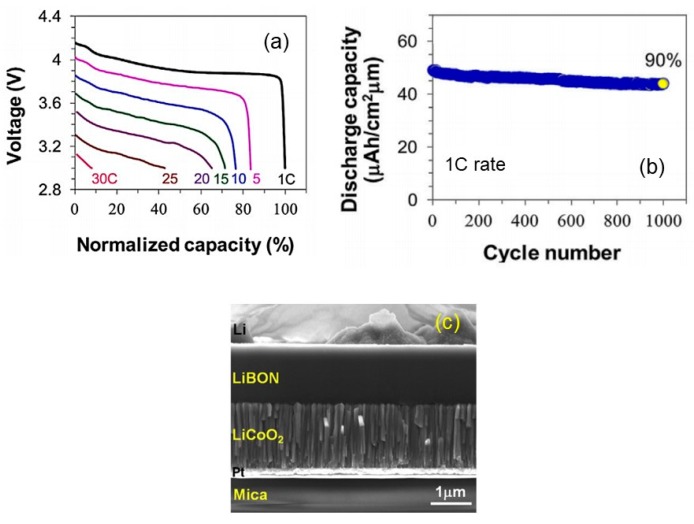
Flexible LiBON-based all-solid state microbattery, Li/LiBON/LiCoO_2_ (**a**) High-rate performance from 1C to 30C. (**b**) Cyclability at 1C and 20 °C. (**c**) SEM cross-section image. Reproduced with permission from [28]. Copyright 2016 Elsevier.

Since 1996, thin films of LiCoO_2_ emerged as promising cathode materials for SSMBs. Several techniques have been employed to grow LiCoO_2_ thin films: Radio-frequency magnetron sputtering (abbreviation rf-sputtering hereafter) [39], spray pyrolysis [40], pulsed-laser deposition (PLD) [41], physical vapor deposition (PVD) [42], etc. In the early work by Wang et al., Li_1.15_CoO_2.16_ (or 0.08 Li_2_O enriched LCO) was prepared using a stoichiometric LiCoO_2_ target in an Ar/O_2_ mixture gas with 3:1 ratio. For film annealed at 600–700 °C, the discharge profiles exhibited the standard potential plateau ~3.9 V with additional two lower capacity plateaus at ~4.2 and ~4.1 V. Over 104 cycles at current density of 100 μA cm^−2^, the capacity fading was 0.0001% and 0.002% for 50- and 500-nm thick thin film cathodes, respectively [39]. More recently, Song et al. [27] investigated the high-rate capability (up to 10C (~0.8 mA cm^−2^)) of Li/Li_2.64_PO_2.81_N_0.33_/LCO/Pt microcells (2 cm × 2 cm × 10 µm) fabricated on a flexible substrates (mica) delivering an initial specific capacity of 39 and 22 µAh cm^−2^ µm^−1^ at a 0.3C and 10C rate, respectively. The high rate and the excellent capacity retention of 95% over 800 cycles are promoted by the (104)/(101) planes of the LCO films. Recently, a bio-compatible flexible lithium-ion thin-film battery was powering the implantable orthodontic system [43,44]. The Si/SiO_2_/Al/LCO:LiPON/Ti microcell (236 μg; 30 µm total thickness) with an unprecedented volumetric energy of 200 mWh cm^−3^ worked over 120 cycles of continuous operation. The flexible SSMB based on the solid electrolyte of lithium boron oxynitride (Li_3.09_BO_2.53_N_0.52_ (LiBON), σ_i_ = 2.3 µS cm^−1^) exhibits impressive high performance from 1C to 30C rate (Figure 4a) demonstrating an initial specific capacity of 49.2 µAh cm^−2^ µm^−1^ at a 1C rate (83.7 µA cm^−2^) with a capacity retention of 90% after 1000 cycles (Figure 4b) [28]. This microcell (10-µm total thickness, 3 cm^2^ surface area) is a stack of a Pt thin layer deposited on a mica flexible substrate, a 1.7-µm thick LCO film deposited by a rf/direct-current hybrid magnetron sputtering technique in 1 Pa of Ar gas, a 1.5-µm thick LiBON film deposited from rf-sputtered 3Li_2_O-B_2_O_3_ target under 0.4 Pa of N_2_ ambient and a 1-µm thick lithium film deposited by thermal evaporation (Figure 4c). Recently, the Li/LiPON/LCO/Au/Ti/SiO_2_/Si was fabricated using a solid electrolyte thin film deposited from Li-rich Li_3_PO_4_ target exhibiting an ionic conductivity of 3.2 × 10^−6^ S cm^−1^ at 25 °C. This microcell delivered specific capacities of 64.5 and 58.0 µAh cm^−2^ µm^−1^ at a current rate of 0.1C and 4C, respectively [29]. Another microcell with the Li/LiPON/LCO/Pt/Ti/TiO_2_/Al_2_O_3_ configuration delivered impressive capacity of 60 µAh cm^−2^ µm^−1^ at a 5C rate without capacity fading after 500 cycles [30]. This superior cycling performance was attributed to the growth process that consisted in the deposition at *T*_s_ = 600 °C without post-treatment. Such thin-film lithium microbatteries was developed as reserve batteries by GS Nanotech Co., Ltd. (Korea) [45].

## 3. Growth of LCO Thin Films

### 3.1. RF-Magnetron Sputtering

**Figure 5:**
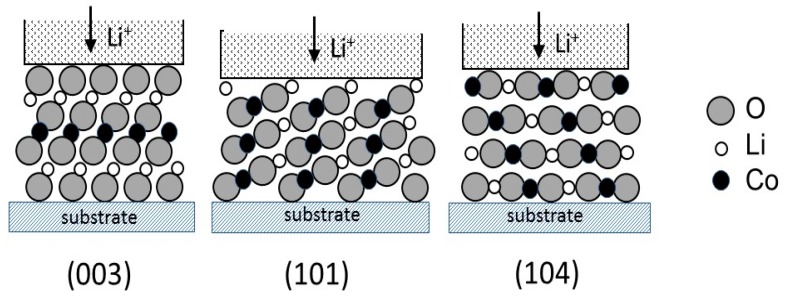
Schematic diagram showing the preferred textures for sputtered LCO films. The close-packed (ccp) (**003**) plane does not allow easy intercalation at the electrolyte-electrode interface, while (**101**) and (**104**) textures favor the easy diffusion path of Li^+^ ions in LCO grains of LCO films.

In this Section, we discussed the influence of the sputtering conditions on the preferential orientations of LCO films, which are generally related to the minimization of the energy during crystal growth. Composition of the Ar/O_2_ gas mixture, gas flow rate, pressure, nature of the substrate, substrate temperature (*T*_s_), deposition rate (*Θ*_dr_), and annealing temperature (*T*_a_) were considered. LCO material being highly anisotropic (lamellar structure), the thin-film deposition process must produce desirable crystalline texture that favors the lithium diffusion at the electrode–electrolyte interface. Typically, there are three structural configurations: (003), (101), and (104) as shown in Figure 5. Due to the layered structure of LCO, the main issue for high electrochemical performance is the orientation of grains. Grain orientation in the (003) direction does not lead to high discharge capacity, in this case Li^+^ ions passed through the grain boundaries. In contrast, (104)- and (101)-oriented grains favor the Li^+^-ion transport. It has been shown that the mechanism responsible for the LCO thin film texture is the volumetric strain energy imposed by thermal mismatch between the film and the substrate during the annealing process [46]. Thus, orientations of grains are dependent of several factors such as the surface state of the substrate and sputter deposition conditions.

The use of an argon atmosphere only results in sputtered films with (003) and (102) planes parallel to the substrate due to the absence of oxygen recombination at the surface. In contrast, the use of a mixture of Ar/O_2_ gas results in dense film and preferred (101) or (104) orientation after subsequent heat treatment. For such films, the structure has an open channel at the interface with the solid electrolyte, which facilitates the Li diffusion from the film surface and lowers the transfer resistance. On the other hand, the growth of LCO films under mixed-gas atmosphere requires less stringent annealing process, i.e., lower annealing temperature of ~300 °C. Trask et al. [47] demonstrated that crystallographic texture of LCO films thicker than 5 µm deposited with an oxygen concentration of 4% in Ar, with a total flow rate controlled to 50 sccm and an operating pressure of 0.5 Pa, shows no detectable (003) peak after annealing at 800 °C for 1 h. Using such conditions, all solid-state microcells (Figure 3) fabricated with a ~15-µm thick cathodes exhibited discharge capacities of 60 µAh cm^−2^ µm^−1^ (600 µAh cm^−2^ as per cathode) at C/10 rate and a capacity retention greater than 95% after 100 cycles at a C/5 discharge rate. Yoon et al. [48] made LCO thin film electrodes on Li_2_O/Al/Si substrates. The use of Li_2_O acts as buffer that suppresses the formation of the Li-deficient phase and avoids the lattice mismatch between LCO and Al (111) plane that was responsible for the growth of (003) plane. By increasing the substrate temperature, the difference in surface energy between the orientations of the atomic planes of LiCoO_2_ is reduced. As a result, the (003)-preferred orientation turns to the (101) one, as observed for 400-nm thick LiCoO_2_ films. This illustrates the strong dependence of the texture on the growth conditions.

Films of lithium cobalt oxide were firstly demonstrated by Wei et al. in 1992 [49], who applied the rf-sputtering method using a crystalline LiCoO_2_ target for smart window application. As-prepared LCO films were nanocrystalline with some (003) out-of-plane texturing due to the substrate temperature held at 300 °C. Films were deposited on various substrates, i.e., tin-indium-oxide coated glass, single crystal of NaCl, and Ni-coated glass, using the following experimental parameters: The target positioned 8 cm below the substrate holder, the sputtering Ar/O_2_ gas mixture of 6:14, rf power of 100 W, *T*_s_ = 300 °C, and *Θ*_dr_ = 0.83 nm min^−1^. Under these conditions, the 200-nm thick films were Li deficient (Li_x_CoO_2−y_ with *x* = 0.4 and *y* = 0.08; Co oxidation state of 3.46) and retained the basic layered α-NaFeO_2_-like structure showing a strong (003) orientation. Since this prior work, substantial efforts have been made over the past decade to prepare LCO thin films by sputtering techniques with well-defined texture and orientation favorable for high electrochemical performance.

The influence of the cathode thickness on the electrochemical properties of SSMBs has been investigated in the range extending from 50 nm to 4 µm [50]. The LCO films were prepared by rf-sputtering in an Ar plasma of 2.7 Pa at the deposition rate of 4–10 nm min^−1^. Oxygen-rich as deposited films (amorphous texture) were subsequently annealed at 700 °C for 2 h for reducing the O/Co ratio. Microcells using a 3-µm thick LiPON film as SSE cycled in the voltage range 3.0–4.2 V showed that shapes of the discharge profiles depend on the cathode thickness *δ*. For *δ* = 50 nm, the lithium equilibration is very fast in the cathode and the discharge capacity of ~1.8 µAh cm^−2^ is almost independent of the current density in the range 2–500 µA cm^−2^. For *δ* = 54 µm, the Li transport becomes very slow in the thick cathode and the specific capacity decreased from 290 to 200 µAh cm^−2^ with the increase of the current density from 20 to 1000 µA cm^−2^, respectively. Despite the lack of texture analysis, it seems that 70% of the maximum capacity for thicker film is due to the non-favorable orientation of the grains in LCO films. Whitacre et al. [51] examined the influence of target aging and deposition geometry on sputtered LCO thin films prepared with target power of 100 W (power density of 2.2 W cm^−2^) under Ar/O_2_ mixed gas (Ar to O_2_ ratio 3:1) with a total gas flow rate of 55 sccm and pressure of 1 Pa. Results showed that these films sputtered from an heavily used target were Li-deficient, while the use of fresh target produced Li-rich LCO films. A ~200-nm thick LCO films deposited on a Si substrate at *T*_s_ = 25 °C from a fresh pre-sputtered target exhibited a strong degree of (104) out-of-plane texture. As a result, caution must be taken to pre-activate the LCO target by pre-sputtering for at least 30 min. In a recent application, Huang et al. [52] patented a sequential method for improving the LCO thin film cathodes that are multilayer films with alternating process conditions. First, sputtering deposition of an LCO layer in Ar/O_2_ gas mixture; second, annealing at a predetermined temperature, at least 300 °C; third, deposition of another very thin LCO film on the annealed one in pure Ar atmosphere, which strengthens the first layer; fourth, depositing a third LCO layer using an Ar/O_2_ mixture. Yoon et al. used a two-step heat treatment to prepare crack-free LCO films using the rapid thermal annealing (RTA) method [30]. It is notable that the (003)-, (104)-, and (018)-plane textures vanish with the increase of *T*_s_, while the (101) plane is not affected. XRD patterns showed that the volume strain energy of the (101) and (104) planes is minimized for thick films (*δ* ≥ 1 µm), which is due to differential thermal expansion between the film and the substrate [53]. The polycrystalline LCO films deposited on the Au/Ti/SiO_2_/Si substrate from the Li-rich Li_1.1_CoO_2_ target and annealed at a moderate temperature of 650 °C showed preferential (101) and (104) plane orientation [54]. Electrochemical tests in the range of 3.0–4.2 V at a current density of 50 µA cm^−2^ displayed a capacity of 58 µAh cm^−2^ µm^−1^ at the 20th cycle with a small capacity fade. LCO films sputtered in 40 sccm argon flow (pressure of 0.2 Pa) on Si/SiO_2_/Ti stack and annealed at 650 °C for 2 h in vacuum exhibited a (104) preferred orientation and the lowest electrical resistivity of 0.37 Ωcm [55].

### 3.2. Electron Cyclotron Resonance (ECR) Sputtering

The Nippon Telegraph and Telephone’s group (Japan) [22,56] used the electron cyclotron resonance (ECR) plasma sputtering method to grow LCO films as cathodes in SSMBs. The authors claimed that this technique, which employs high-energy ionic radiation, could produce well-crystallized LiCoO_2_ films without a post-heating treatment by adopting favorable deposition conditions. ECR plasma, which includes Ar and O_2_ gas flow is generated by introducing microwaves (2.45 Ghz) into a magnetic field. In the first investigation, the LCO films were prepared from sputtered Li_x_CoO_2_ targets with Li-enriched composition in the range 1.0 ≤ *x* ≤ 2.0. The sputtering process (microwave and rf powers of 800 and 500 W, respectively) was carried out at *T*_s_ = 300 °C under fixed Ar/O_2_ gas ratio of 40:1 with a total pressure of 0.14 Pa. The 3.7-µm thick cathode films deposited on a 500-nm thick Pt layer showed a pure HT-LiCoO_2_ phase when obtained for the Li-rich target (*x* = 2). Such a thin film electrode exhibited good electrochemical properties, i.e., 68 µAh cm^−2^ µm^−1^ at 0.2 mA cm^−2^ discharge current. The 6.2 µm thick films similarly produced were utilized in Li/LiPON/LCO/Pt/Ti/quartz glass SSMBs that provided a discharge capacity of about 250 µAh cm^−2^ (40.3 µAh cm^−2^ µm^−1^) with good cycle ability [22]. This low specific capacity was attributed to the presence of Co_3_O_4_ impurities and the (003) out-of-plane texture, due to the absence of post-treatment.

## 4. Influence of Preparation Conditions

A good knowledge of the relationship between the process parameters and the resultant structure of the film is required to obtain the desired properties of the deposit [57,58,59,60,61,62,63,64,65,66,67,68,69,70]. The monitoring of many rf-sputtering parameters, i.e., sputter power, working atmosphere, working pressure, target-substrate distance (*d_st_*), and deposition temperature (*T*_s_), allows us to control the texture, orientation and crystallinity of LCO thin films. RF sputtering improved the density and homogeneity of thin films that favor the low thin-film resistance. Currently, SSMBs are fabricated on a rigid substrate, i.e., silicon, alumina, or on a flexible substrate, i.e., mica. A polyimide (pyromellitimide-1,4-diphenyl ether made by Dupont under the trade-name Kapton) was also used as a cell supporting substrate [57]. LCO cathode thin films are deposited on a thin noble metal, i.e., Pt [58], Au [69], Ag [60], Cu foil [61], or Al layer [62], which acts as the current collector; both films are deposited on the top of a SiO_2_ layer, which electronically insulates the electrode from the silicon wafer. Sometimes, an additional Ti layer (30-nm thick) is deposited to enhance the adhesion of Pt to the SiO_2_ surface. Table 3 lists the various experimental conditions taken from the literature for the preparation of LCO thin films deposited by rf-sputtering technique along with the main electrochemical performance. According the literature, typical RF sputtering deposition of LCO thin films is realized under the following experimental conditions: The radio frequency is 13.56 MHz, the target-substrate distance is *d_st_* = 50–80 mm, and the sputtering power density varies from 1.2 to 5.0 W cm^−2^.

**Figure 6:**
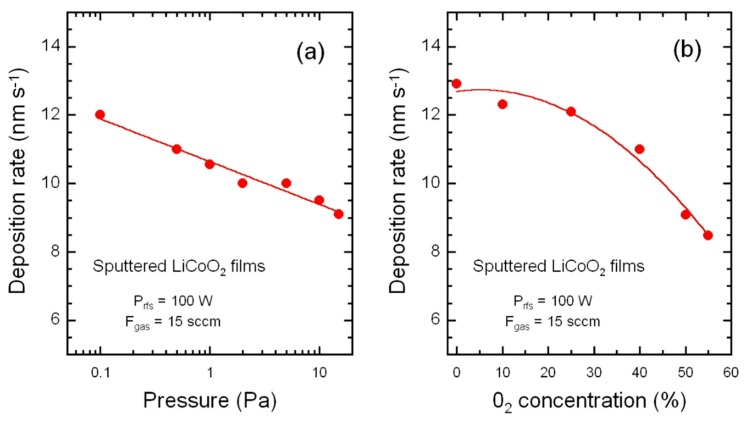
Evolution of the sputtering deposition rate of LCO films as a function of the working pressure (**a**) and the O_2_ concentration in the Ar/O_2_ gas mixture (**b**).

However, some precautions must be taken. For obtaining good quality LCO thin films with adequate surface chemical composition and morphology that dictate their electrochemical performance, it is necessary to pre-sputter the virgin target for at least 2 to 3 h at high rf-power to eliminate the surface contamination. To avoid lithium deficient LCO film due to sputtering over a prolonged period [51], Kusuril proposed the use of a powder target rather than a solid one, i.e., pressed and sintered pellet of ceramic powder and binder material [61]. The deposition rate is also an important parameter for the growth a stoichiometric LCO thin films. This factor was first established by Bates et al. [46] reporting the mechanism for the preferential orientation of LCO grains grown on a (100)Si/Co/Pt substrate. By combining the deposition rates in the range 1–10 nm min^–1^ and the deposition temperature in the range 50–300 °C, different degrees of (003), (101), and (104) orientations were determined. It was also mentioned that at high substrate temperature, due to the increase of the surface mobility of adatoms, the texture changed from (101)–(104) to (003). As shown in Table 3, one observes a wide range of *Θ*_dr_ values using by the researchers. Nevertheless, it appeared that *Θ*_dr_ varies with both the working pressure (Figure 6a) and the concentration of oxygen in the Ar/O_2_ gas mixture (Figure 6b).

### 4.1. Influence of the Substrate

LiCoO_2_ thin films have been prepared on varieties of substrates (see Table 2), the most popular being (100)-oriented silicon. However, to avoid the reaction of Si with LCO (because Si forms an alloy with Li), a thin layer of SiO_2_ and a metallization is formed (see Figure 3). Lee et al. [72] studied the influence of the substrate, i.e., sintered alumina and SiO_2_/Si (100) substrates, on the microstructure of sputtered LCO thin films. Film were deposited at the rate of 0.9–1.2 nm min^−1^ in the presence of 0.5 Pa Ar/O_2_ mixture gas (9:3 ratio) flowing at 12 sccm and post-annealed at 800 °C in O_2_ atmosphere for 30 min. For both substrates, a 300 nm-thick layer of Pt was deposited as the current collector.

**Figure 7:**
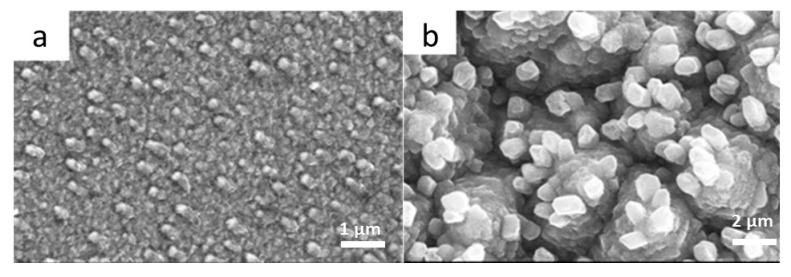
SEM images of as-sputtered LCO thin films deposited (**a**) on polished Si substrate and (**b**) on textured Si substrate obtained by chemical etching. The film (**b**) consists of layered like grains mostly distributed as individual clusters composed of few numbers of rough grains, which were vertically agglomerated. Reproduced with permission from [75]. Copyright 2014 Elsevier.

LCO films deposited on Al_2_O_3_/Pt substrates displayed a rough surface with several cracks induced by thermal expansion of the substrate, while films deposited on Si/SiO_2_/Ti/Pt substrates consisted of small grains without cracks. Such films exhibited the specific capacity of 27 µAh cm^−2^ µm^−1^ at a current density of 50 µA cm^−1^ after 150 cycles with an average capacity decrease rate of 0.05% per cycle. Jeevan-Kumar et al. [73] produced LCO thin films (1.8 µm thick) from a Li-enriched (10%) sintered 3-in LiCoO_2_ target to compensate the volatility of Li. These films deposited on metallized Si substrate kept at *T*_s_ = 250 °C with subsequent annealing at 650 °C under an oxygen pressure of 5 Pa showed a well-oriented HT-LiCoO_2_ phase with predominant (104) planes. The high c/a ratio of 4.997 characterized the layered (*R*-3*m*) structure. Jeong et al. [74] interposed a thin Al_2_O_3_ layer (10 nm thick) deposited at the rate of 3.5 nm min^−1^ between the LCO film cathode and the LiPON film electrolyte, which decreases the interfacial resistance owing to the formation of a solid solution LiCo_1−y_Al_y_O_2_ at the interface during the heat treatment at 400 °C for 5 h. The same group identified the importance of substrate texturing and the formation of LCO film avoiding post-deposition treatment. The morphology of the LCO thin-film deposited on the Au/Ti/SiO_2_ stack was investigated as a function of the substrate texture using polished Si and textured Si (obtained by chemical etching using the H_2_O:HCl:H_2_O_2_ (6:1:1) solution; Figure 7). Surprisingly, the films deposited on textured Si having (003)-oriented grains (as per XRD patterns) delivered better capacity retention than the (104)-oriented films grown on polished-Si substrate. However, the film deposited on polished Si and heat-treated at 650 °C displayed the high initial discharge capacity of 60 µAh cm^−2^ µm^−1^ [75]. Taking the advantage of the direct deposition on a metal substrate acting as the current collector, similar behavior was obtained on stainless-steel (STS304) foil. Despite its cost, the Au current collector film can favor the preferred orientation of the LiCoO_2_ thin film cathode. A comparison between textured and annealed STS304 was established [66]. Using an Ar/O_2_ mix gas of (4:1), the substrate having a strong (220) texture produces LCO films with a (003) preferred orientation, while the film deposited on annealed STS304 exhibited the (104) preferred orientation. As a result, the initial discharge capacity of the film deposited on the annealed stainless steel was higher than that of the film deposited on the textured substrate, but reverse behavior was observed for the LCO cathode cyclability. When Al foil is used as substrate, the degree of crystallization of the sputtered LCO films varies with the thickness of the Al coating deposited on the Al foil. The thinner the Al coating, the faster the crystallization process [63].

### 4.2. Deposition Conditions

In this section, we examined the relationship between the structure and morphology of LCO films and the experiments conditions, i.e., substrate temperature, deposition rate, sputter power, working pressure, substrate bias, and post-annealing process. The use of a heated substrate rather than a deposition at room temperature has been a debating issue. In most cases, the films deposited at *T*_s_ = 25 °C were found to be amorphous and subsequently developed a (003), (101), (110), or (104) out-of-plane texture upon annealing, depending on thickness [39,49]. Stockhoff et al. [14] stated that a lattice structure similar to the LT-LiCoO_2_ phase is obtained at *T*_s_ < 300 °C, while HT-LiCoO_2_ is prepared at *T*_s_ > 300 °C and in the Ar/O_2_ (3:2) atmosphere. LCO films were grown on Pt-coated Si wafers heated in the range 25–600 °C in an Ar/O_2_ (ratio 3:1) gas flow rate of 12 sccm. Films deposited at 250 °C and annealed at 600 °C showed strong (104) orientation. An electrochemical test carried out under a current density of 10 µA cm^−2^ in the potential range of 4.2–3.0 V displayed responses depending on preparation conditions. Specific discharge capacities of 50, 42.5, and 30 µAh cm^−2^ µm^−1^ were obtained after 10 cycles, for films 25 °C-deposited and annealed at 600 °C, 250 °C-deposited and annealed at 600 °C and 600 °C-deposited, respectively [70]. The structure of LCO films deposited on Pt/Ti/quartz glass at different substrate temperatures in the range 25–500 °C changed from amorphous to strongly oriented (003) texture. The films grown at *T*_s_ = 400 °C displayed the well-defined discharge voltage plateau of crystallized LCO at 3.9 V and delivered an initial specific capacity of 54.5 µAh cm^−2^ µm^−1^ [76]. The multilayer system Si(substrate)/Si_3_N_4_/TiO_x_/Pt/LCO has been fabricated, in which Si_3_N_4_ is used as a barrier against the Li diffusion into silicon and TiO_x_ is an adhesion layer with an optimum thickness of 25–45 nm [77]. Note that SiO_2_ between LiCoO_2_ and Si can work as a solid-state electrolyte allowing transport of Li ions and trap Li ions when external voltage is removed thus increasing device retention. Recently, Hu et al. investigated the effect of the SiO_2_ thickness on the properties of the Pt/LiCoO_2_/SiO_2_/Si stacks demonstrating the Li trapping mechanism [78].

The gravimetric density and the porosity of films are important parameters playing a major role for the transport of Li ions in the cathode material. Experimental results have evidenced the dependence of the density on the sputter pressure and film thickness [59,79]. The film density is currently determined by fitting the X-ray reflectivity measurements. Ziebert at al. [59] reported an increase of the density from 4.34 to a maximum value of 4.75 g cm^−3^ for increasing pressure from 0.15 and 1 Pa, respectively for 3-µm thick LCO films. At pressure of 10 Pa, the density strongly decreased to 3.5 g cm^−3^. SEM images displayed visible gaps and voids, giving evidence of an increase of the porosity. For 100-nm thick films, the decrease of the density is less dramatic, at 10 Pa a value of 4.2 g cm^−3^ was measured.

Due to the difference of the atomic weights between Li and Co, the Li/Co ratio is sensitive to the rf power [58]. LCO films with Li/Co close to 1 was obtained with a small fraction of O_2_ in the mixed gas (≤25%) under working pressure of 2 Pa using a sputter power in the range 75–100 W. Higher sputter power produces a reduction of the compositional Li/Co ratio. Pan and Yang reported the effects of the radio-frequency sputtering powers (80–200 W) on the micro-structures and electrochemical properties of LCO thin film electrodes. LCO films, grown on Pt-wafer substrates kept at 55 °C and at high sputtering power of 200 W, had (101)-oriented with big grains of 150 nm in size and exhibited a specific capacity of 61 µAh cm^−2^ µm^−1^ when discharged at a constant current of 20 µA cm^−2^ between 4.2 and 3.0 V [68]. Similar studies were carried out by Jeon et al. [80] showing a specific capacity of ~59 µAh cm^−2^ µm^−1^ taken at 30 µA cm^−2^ for the LCO films deposited on stainless-steel substrate at 150 W sputtering power in an Ar/O_2_ mixture of 9:1. Pracharova et al. [81] stated that neither the substrate temperature nor the substrate material influences the Li/Co atomic ratio in LCO thin films deposited on Si/SiO_2_/Ti/Au substrate, while the radio-frequency discharge power is an important parameter to control the stoichiometry. For a sputter power of 60 W (i.e., 3.06 W cm^−2^), the films were stoichiometric but an unfavorable (003) orientation was observed by X-ray diffraction and Raman spectroscopy. Using a low power of 25 W, Rao et al. [82] prepared out-of-stoichiometric Li_x_CoO_y_ films with *x* *<* 1 and *y* *>* 2. Electrical conductivity measurements showed that the film Li_0.8_CoO_2.7_ is metallic, which can be beneficial for its role as a cathode material but contains Co_3_O_4_ impurity phase, which is detrimental.

**Figure 8:**
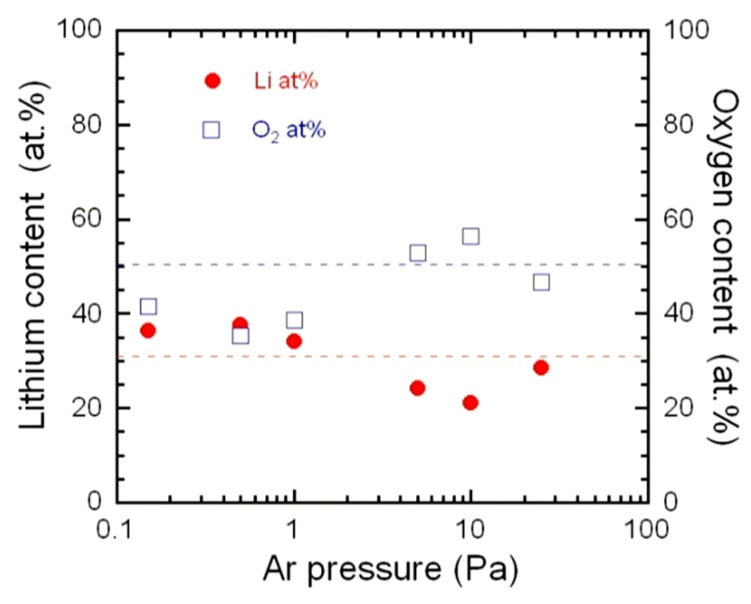
Variation of the lithium and oxygen content as a function of the Ar pressure for LCO thin films deposited on (100)-oriented Si substrates. Reproduced with permission from [59]. Copyright 2010 Elsevier.

The effect of the sputter gas mixture on the LCO thin film composition has been widely demonstrated. For sputter gas consisting of 100% Ar, the Li/Co ratio was found to be 0.88 [40] or 1:0 ± 0.1 [83]. Park et al. [84] studied the influence of the pressure (from 0.4 to 2.4 Pa) on the LCO thin film properties and reported the highest discharge capacities for 2.4 Pa but did not test higher pressures. Ziebert et al. [59] investigated the composition of LCO thin films sputtered in Ar atmosphere. Figure 8 presents the variation of the lithium and oxygen content as a function of the Ar pressure in the range 0.15–25 Pa. Elemental analyses carried out by inductive coupled plasma (ICP) show lithium excess for deposition at low pressures (*P*_Ar_ ≤ 1 Pa) and oxygen deficiency in the range 0.5–1.0 Pa. An annealing treatment in Ar/O_2_ (4.5:5) atmosphere of 10 Pa at temperatures between 100 and 600 °C for 3 h compensate for the oxygen deficiency.

For oxygen-rich Ar/O_2_ mixes of 1:10, 1:2, or 1:1, the Li/Co ratio was reported to be 0:8 ± 0:08 (O/Co = 2.7) [85], 0.4 (O/Co = 1.92) [49], or 1:15 ± 0:02 (O/Co = 2.2) [39], respectively. Bouwman et al. [69] prepared submicrometer LCO films deposited on (100)-oriented Si substrate using a stoichiometric target in rf-sputtering conditions of *T*_s_ = 300 °C, with background pressure of 0.7 Pa for the Ar/O_2_ mixture in 3:1 ratio. At a growth rate of 0.5 nm min^−1^ and after annealing at 600 °C for 3 h, the films were preferentially oriented with their (110) planes parallel to the substrate surface. However, an O/Co ratio of 2.02 was reported. The use of only argon in a conventional sputtering process may create cracks in LCO films. In thick films, distinct columnar structures appeared after annealing, which must be eliminated, because they result in lower film density and formation of cracks limiting the lithium diffusion. Mixing O_2_ with Ar during the deposition allows the fabrication of thick films with better morphology, good stoichiometry, and battery performance as a result of the presence of oxygen improving the film nucleation and growth. Park et al. [84] investigated the influence of sputtering gas pressure on the 200-nm thick LiCoO_2_ thin films prepared from a 4 inch target sputtered at 200 W under operating Ar/O_2_ gas ratio of 8:2 maintained at pressure in the range of 0.3–1.8 Pa. Films exhibiting the best performance were annealed at a temperature that does not exceed 400 °C. The higher value of specific capacity (ca. 38 µAh cm^−2^ µm^−1^) is obtained for film grown at pressure of 1.3 Pa. The matter transport through Ar plasma in LCO thin films sputtering was analyzed using the Monte Carlo method [81]. Stable Li/Co ratios have been obtained at 5 Pa pressure and for the substrate-target distance in the range of 5−11 cm.

**Figure 9:**
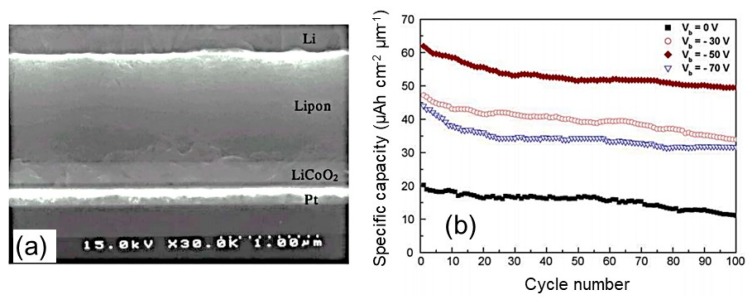
(**a**) Cross-section image of all solid-state Li/LiPON/LCO/Pt microbattery. (**b**) Cyclability as a function of the substrate biases used during the rf sputtering process of the LCO cathode film. Reproduced with permission from [86]. Copyright 2005 Elsevier.

Among the various ways of improvement of the growth process of crystalline LCO thin films, the use of a substrate bias has been proposed as the key parameter for decreasing the use of annealing treatment. The influence of the substrate bias on the LCO film crystallinity was recognized since 2005. The effect of substrate biases (*V*_b_) in the range 0–100 V has been investigated on the morphology and electrochemical performance of LCO thin films (350 nm thick) deposited on Si(100)/SiO_2_/Ti/Pt under Ar/O_2_ (45:5) gas flow at a working pressure of 0.5 Pa using a sputtering power of 200 W [86]. At *V*_b_ = 0 V, the XRD patterns showed tiny (003) and (006) peaks that disappeared with application of *V*_b_ in favor of the (101) Bragg line. The LCO films deposited at *V*_b_ = −50 V had an average grain size of 4.9 nm (density of 5.16 g cm^−3^), while at *V*_b_ = −70 V the presence of Co_3_O_4_ was detected. The charge–discharge tests carried out at a 1C rate in the voltage range of 3.0–4.3 V displayed a specific capacity of 60 µAh cm^−2^ µm^−1^. The cycling performance of a Li/LiPON/LCO/Pt solid-state microbattery is shown in Figure 9. The −50 V-biased electrode showed a good capacity retention of 90% after 100 cycles.

In 2011, Navone et al. [24] lowered the annealing temperature to 500 °C by the optimization of bias sputtering at −50 V (with Ar/O_2_ ratio 3:1) that produced 0.5-µm thick crystalline deposits without any trace of the cubic phase. Such films delivered a specific capacity of 50 µAh cm^−2^ µm^−1^ after 140 cycles at 10 µA cm^−2^ current density. Next, the same group of research recognized that the crystallinity is not only controlled by the annealing temperature and the bias voltage but also by the pressure of the gas mixture. LCO films deposited by RFS onto Al substrates and post-annealed treated at 500 °C in air for 2 h show various morphologies and structures as a function of the operating Ar/O_2_ gas pressure ranging from 0.55 to 3 Pa. Variation of gas mixture, gas pressure, substrate bias promoted the relative amount of LCO phase mixture, i.e., mixture of the layered HT-LCO and cubic LT-LCO.

Optimized films were produced at 3 Pa in an Ar/O_2_ gas mixture of 3:4 delivering a high specific capacity of 67 µAh cm^−2^ µm^−1^ at C/5 rate but these studies were carried out in a liquid electrolyte only [87]. Taking the advantage of a high-rate bias, LCO films were tested in a non-aqueous electrolyte cell [88] and, finally, a rf-sputter-deposited microbattery LCO/LiPON/Li was fabricated on the Si/SiO_2_/Sn_3_N_4_/Ti/Au substrate that delivered an initial specific capacity of 49 µAh cm^−2^ µm^−1^ at 10 µA cm^−2^ current density and showed a capacity retention of 82% after 800 cycles [25].

Annealing is requested for the formation of well-crystallized films with preferential (104) or (101) orientation. Sufficient annealing temperature is also especially important in order to get the HT-LCO phase that avoids poor cyclability. However, heat treatment at temperature as low as 500 °C could be preferable to maintain a good adhesion on the substrate. The drawbacks of annealing at temperatures greater than 700 °C producing cracks and voids, i.e., micro-short paths, has been also mentioned [86,89]. The combination of a substrate temperature of 250 °C and annealing process under O_2_ ambient at 650 °C leads to LCO films with the characteristic (104)-preferred plane [90]. Rapid thermal annealing (at 650 °C for 15 min was proposed. LCO films were grown on Pt-coated Si wafers heated in the range 25–600 °C in an Ar/O_2_ (ratio 3:1) gas flow rate of 12 sccm. Films deposited 250 °C and annealed at 600 °C showed strong (104) orientation. An electrochemical test carried out under a current density of 10 µA cm^−2^ in the potential range of 4.2–3.0 V displayed responses depending on preparation conditions. Specific discharge capacities of 50, 42.5, and 30 µAh cm^−2^ µm^−1^ were obtained after 10 cycles, for films 25 °C-deposited and annealed at 600 °C, 250 °C-deposited and annealed at 600 °C and 600 °C-deposited, respectively [70]. Well-crystallized sputtered LCO films (500 nm thick) were obtained after annealing at different temperatures (400–700 °C) for 1 h in O_2_ ambient. The films were deposited at the rate of 0.08 nm s^−1^ at room temperature on NASICON-type electrolyte Si- and Ge-doped Li_1.3_Al_0.3_Ti_1.7_(PO_4_)_3_ (LATP) substrates in a working Ar atmosphere kept at 0.5 Pa [26]. It appeared that films displayed large voids and a triggered nucleation of a face-centered cubic (fcc) phase for annealing at 700 °C. Films annealed at 500 °C showed strong crystallographic (101) texture with layer planes aligned nearly normal to the substrate. Electrochemical tests of the cell Li/LiPON/LATP/LCO/Pt, where 1-µm-thick LiPON acts as a buffer layer to avoid degradation of the LATP in contact with Li anode, were carried out between 3.3 and 4.2 V at a 0.01C rate. This cell exhibited an initial specific discharge capacity of 40 µAh cm^−2^ µm^−1^ that decreased to 15 µAh cm^−2^ µm^−1^ after 50 cycles. Xie et al. [91] show that amorphous LCO films deposited on NASICON glass ceramics Li_1+__x__+__y_Al_x_Ti_2__−__x_Si_y_P_3__−__y_O_12_ (LATSP) at *T*_s_ < 180 °C. An initial discharge capacity of 210 mAh g^−1^ was delivered by as-deposited LCO thin films (0.5 µm thick) on LATSP in Ar/O_2_ (7:3) at a power of 100 W. A rapid-thermal annealing (RTA) process at 700 °C under flowing oxygen gas (for 20 min) was shown to be a successful method to obtain LCO thin films that consist of grains with (101) and (104) preferred orientations [92].

## 5. Electrochemical Properties of Sputtered LCO Films

### 5.1. Charge–Discharge Behavior

The maximum capacity (in Ah) of any lithium cell is largely determined by the amount of active material in the positive electrode. The specific capacity *Q*_th_ in ampere-hour per mass (Ah·kg^−1^), or equivalently in mAh·g^−1^ is obtained from the Faraday law [93]:

(1) $$Q_{th}=\frac{1000\times nF}{3600\times M_{w}}=\frac{26.8}{M_{w}}\times n$$

where *M*_w_ is the molecular mass of the “limiting” electrode material. With the transfer of *n* = 1e^−^ per formula unit, the theoretical specific capacity of LiCoO_2_ (*M*_w_ = 97.87 g mol^−1^) is 273.8 mAh·g^−1^. Note that the reversible capacity, however, is limited to 140 mAh g^−1^ when LiCoO_2_ is cycled between 3 and 4.2 V, corresponding to extracting and inserting about 0.5 Li per LiCoO_2_.The relation between the gravimetric capacity, *Q_m_*, of the material, and the volumetric capacity of a film, *Q_f_*, is given by:

*Q_f_* = 0.36 *d Q_m_* (2)

where *Q_m_* is expressed in mAh g^−1^, *Q_f_* is translated in µAh cm^−2^ µm^−1^ (or mC cm^−2^ µm^−1^), and *d* is the density of the material in g cm^−3^. With a density of 5.06 g cm^−3^, the theoretical volumetric capacity of LiCoO_2_ is 137.8 µAh cm^−2^ µm^−1^ if porosity is ignored. Decreasing the film thickness implies a large electrode area or a limited capacity. A thin electrode film exhibiting high intercalation rates is expected to have a short diffusion pathway *L* for the Li^+^ ions according to Fick’s law. In case the chemical reaction proceeds by a single-phase process, i.e., within a solid solution, the characteristic time *τ*_sp_ for Li^+^ ions to reach the surface of any active particle of dimension *L* is given by:

(3) $$\tau_{sp}=\frac{L^{2}}{4\pi D_{Li}}$$

where *D*_Li_ is the chemical diffusion coefficient of moving ions in the host framework. However, Wang et al. [39] reported that thick electrodes (>1 μm) could deliver higher current pulses. In this case, the preferred texture of the film plays a major role at the electrolyte–electrode interface. The electrochemical behaviors of sputtered thin-film LCO cathode were characterized under high-voltage conditions in microbatteries using either an organic electrolyte (1 mol L^−1^ LiPF_6_ in ethylene-dietylene carbonate) or a LiPON solid film electrolyte. One of the first attempts show that, even annealed at 600 °C in air, the 0.2-µm thick LCO films deposited with a low power of 50 W on SnO_2_-coated glass under working pressure 0.7 Pa have only delivered a discharge capacity of 114 mC cm^−2^ µm^−1^ (in the voltage range 4.1–3.0 V) due to the mixed LiCoO_2_ + Li_1.47_Co_3_O_4_ crystalline phase [94]. Liao and Fung [63] obtained a first discharge capacity of ~42, ~50, and ~61 µAh cm^−2^ µm^−1^ at a discharge rate of 10 µA cm^−2^ in the potential range 4.25–3.0 V for LCO film (1.3 µm thick) annealed at 500, 600, and 700 °C for 2 h, respectively. These films originally deposited on Pt/Ti(20 nm)/SiO_2_(600nm)/(100)Si substrate heated at 250 °C under *P*_O_2__ = 0.5–5.0 Pa (gas flow rate of 12 sccm, power of 100 W and *d_st_* = 40 mm) had a nanocrystalline structure with (104) out-of-plane orientation.

**Figure 10:**
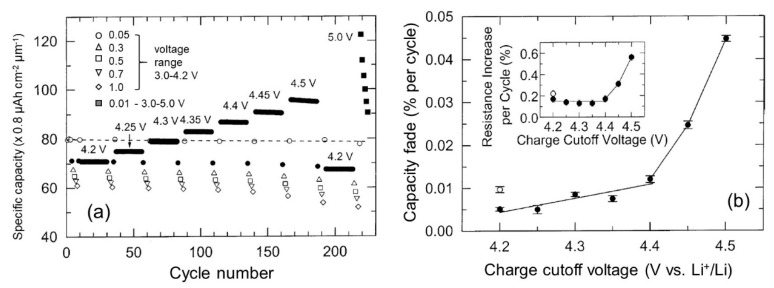
(**a**) The variation of the discharge capacity of sputtered LCO thin films with the charge cutoff voltage for a discharge voltage limit fixed at 3.0 V and a current density of 0.1 mA cm^−2^ (black dots). The black squares are the capacities of cell cycled at 10 µA cm^−2^ in the potential range 3.0–5.0 V. Other opened symbols correspond to different discharge currents (in mA cm^−2^) in the potential range 3.0–4.2 V. (**b**) Capacity fade per cycle as a function of the charge voltage cutoff. Inset presents the increase in cell resistance per cycle. Reproduced with permission from [95]. Copyright 2003 Elsevier.

Taking into account the advantage of the LiPON stability up to 5.5 V vs. Li+/Li, the galvanostatic charge-discharge (GCD) measurements were carried out in the voltage range of 3.0–5.0 V at a current density of 10 µA cm^−2^ [95]. Figure 10a shows the variation of the discharge capacity with the charge cutoff voltage for a discharge voltage limit fixed at 3.0 V. The 1.2-µm thick LCO film can sustain a capacity of ~85 µAh cm^−1^ µm^−1^ (170 mAh g^−1^), which correspond at *x* = 0.63 Li extracted when the cell is charged at 4.4 V. However, upon cycling to further voltage (>4.4 V), a two-phase reaction (CoO_2_ and Li_x_CoO_2_ phases) associated with the variation of the c-lattice parameter of 3.2% induced an increase of cell resistance and capacity fades (Figure 10b). The formation of cracks appeared for the LCO film is cycled to 5 V due to the overcharge process.

Noh et al. compared the microstructure and electrochemical performance of sputtered LiCoO_2_/LiNiO_2_ multilayer thin film cathode with that of LiCoO_2_ single-layer [96]. Using an Ar/O_2_ gas mixture (4:1) at a flow rate of 150 sccm with a pressure maintained at 0.3 Pa, both electrodes deposited at *T*_s_ = 65 °C had the (003)-preferred orientation. The initial discharge capacity of the multi-layer electrode was ~53 µAh cm^−2^ µm^−1^ at a current density of 10 µA cm^−2^ in the potential range of 3.0–4.2 V, approximately 30% larger than the single LCO electrode. Via in situ measurements, Cho et al. [71] investigated the thermal conductivity of sputtered Li_x_CoO_2_ films electrochemically delithiated in the range 1.0 ≤ *x* ≤ 0.6. LCO films (500-nm thick) were deposited by reactive sputtering at a rate of 0.8 nm min^−1^ on *c*-plane oriented sapphire substrates coated with ~100 nm of SiO_2_ and an ~80 nm Al layer as current collector. They were randomly textured after an annealing process at 500 °C in air. During delithiation, the thermal conductivity decreases reversibly (from 5.4 to 3.7 W m^−1^ K^−1^) and the elastic modulus decreases as well from 325 to 225 GPa.

### 5.2. Li^+^ Ion Diffusion

The Li-ion chemical diffusion coefficients, *D*_Li_, were measured using various electrochemical methods, i.e., cyclic voltammetry (CV), galvanostatic intermittent titration technique (GITT), potentiostatic intermittent titration technique (PITT), and electrochemical impedance spectroscopy (EIS). In CV measurements, *D*_Li_ is determined from the Randles–Sevcik relation, which describes the influence of the sweep rate on the redox peak current, while other methods evaluate the composition dependence of *D*_Li_. The PITT and GITT methods rely on solving Fick’s laws the variation of current (voltage) vs. time is measured after application of a potential (current) step to slightly modify the electrode composition of the electrode. In EIS measurements, the composition dependence of *D*_Li_ is estimated from the Warburg impedance related with the straight line of 45° slope of the Nyquist diagram. The *D*_Li_ values are considered to be more reliable when obtained from GITT and PITT methods [67]. However, the disparate values of *D*_Li_ are often due to a poor evaluation of the actual surface area of the electrode. It is recommended to use Brunauer–Emmett–Teller (BET) surface measurements.

**Figure 11:**
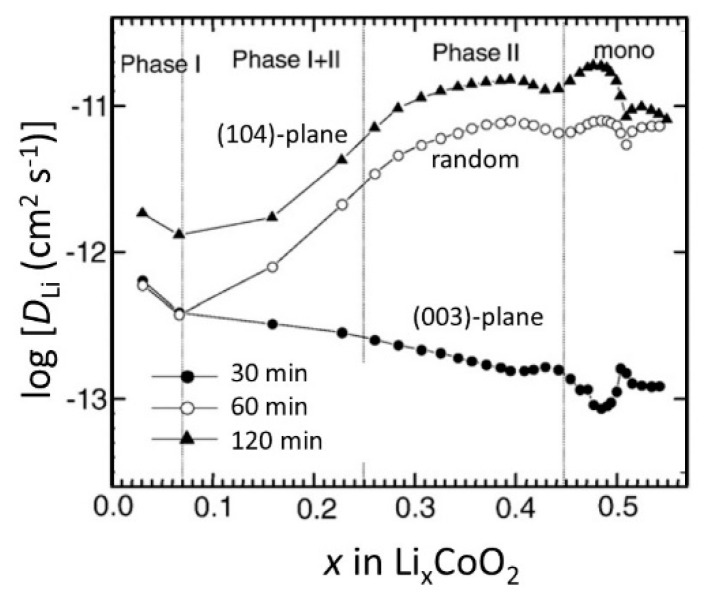
Compositional dependence of the Li^+^ ion diffusion coefficients in LCO thin films sputtered at different durations, as measured using the potentiostatic intermittent titration technique (PITT) method. Reproduced with permission from [79]. Copyright 2008 Elsevier.

The influence of the diffusion plane orientation on electrochemical properties of thin film LCO electrodes has been pointed out by several authors [67,79,97]. Submicrometer LCO films deposited on Si substrates exhibited a strong *a*-axis orientation, which favors the intercalation rate and cycling efficiency. However, Bouwman et al. reported an intercalation rate hindered by a large charge-transfer resistance and phase boundary motion rather than the diffusion-limited behavior currently observed in LCO crystal [97]. Xie et al. [67,79] reported the Li^+^ ion kinetics in three LCO thin films sputtered for different duration (*t*_d_) of 30, 60, and 120 min on polished Al_2_O_3_ substrates covered with a 900 nm thick Au layer. A 0.31-μm thick LiCoO_2_ thin film (sputtered for *t*_d_ = 30 min) showed a high (003) orientation, while a 1.35-μm thick film (sputtered for *t*_d_ = 120 min) exhibited high (104) orientation. CV measurements showed that the peak current (*I*_p_) follows a linear relationship with the square root of the scan rate (ν^1/2^) indicating a diffusion-controlled process. Thus, *D*_Li_ can be calculated using the Randles–Sevcik relation *I*_p_ = *f*(ν^1/2^):

(4) $$D_{Li}=\frac{5RT}{n^{3}F^{3}A^{2}C_{Li}^{2}}\frac{I_{p}^{2}}{\nu }$$

where *R* and *F* are the usual constants, *T* is the absolute temperature, *A* is the surface area of the electrode, and *C*_Li_ is the concentration of Li in the electrode. The (104)-oriented film exhibited a larger *D*_Li_ value of 7.7 × 10^−12^ cm^2^ s^−1^ than 6.4 × 10^−13^ cm^2^ s^−1^ for the (003) oriented thin film. Note that these values are “apparent” diffusion coefficients because the compositional dependence of *D*_Li_ cannot be determined by the CV technique. However, the PITT method based on solving the Fick’s diffusion equation determines *D*_Li_ as a function of *x*(Li) in Li_x_CoO_2_ by recording the time dependence of the transient current (*I*_t_) when a potential step is applied to the film:

(5) $$D_{Li}=\frac{dLn(I_{p})}{dt}\frac{4L_{}^{2}}{\pi^{2}}$$

where *L* is the thickness of the film. The compositional dependence of *D*_Li_ calculated using Equation (5) is presented in Figure 11, which displays the structural domains of the LiCoO_2_ electrode according the phase diagram reported by Bouwman et al. [97].

**Figure 12:**
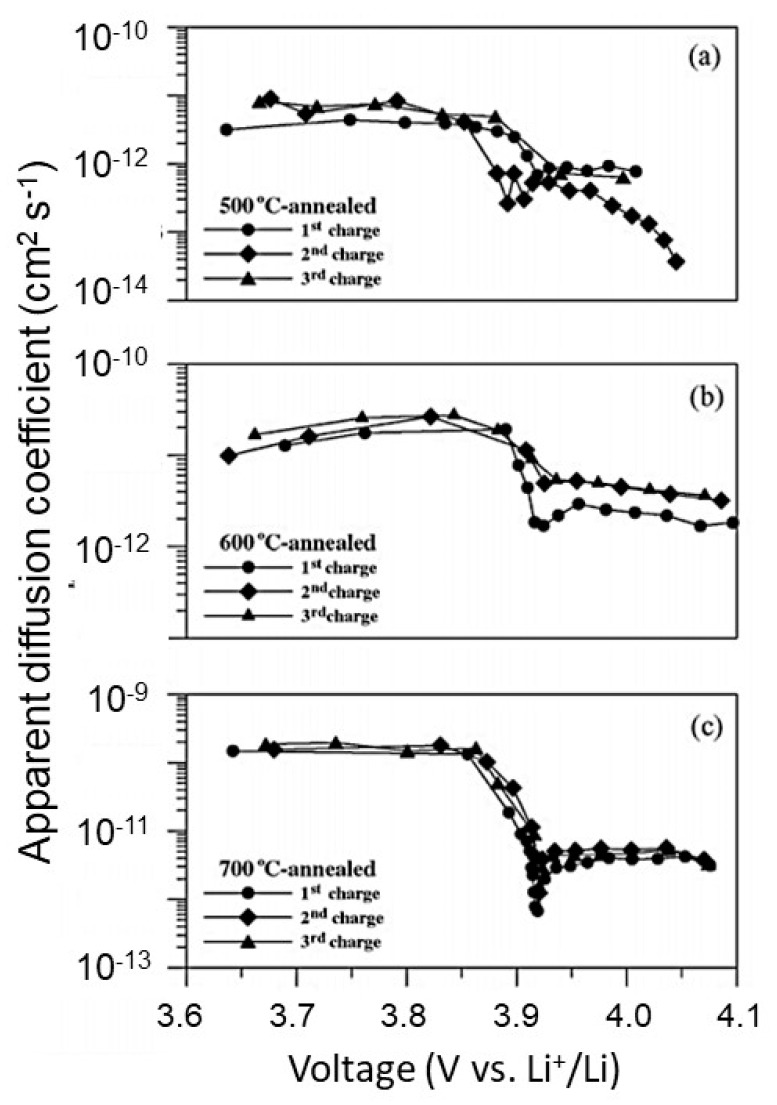
*D*_Li_ as a function of cell voltage obtained from different charging cycles from the galvanostatic intermittent titration technique (GITT) for (**a**) 500 °C-annealed, (**b**) 600 °C-annealed, and (**c**) 700 °C-annealed HT-LiCoO_2_ films. Reproduced with permission from [58]. Copyright 2007 Elsevier.

Liao et al. [58] investigated the effect of various rf-sputtering parameters on the Li^+^ ion diffusion coefficient if LCO films deposited on Pt-coated Si wafers. The 700 °C-annealed film showing good crystallinity with (104) preferred orientation exhibit a high discharge capacity of 61 and 56 µAh cm^−2^ µm^−1^ at a discharge rate of 10 and 50 µA cm^−2^, respectively, while the 500 °C-annealed film displays a discharge capacity of 35 µAh cm^−2^ µm^−1^ at a discharge rate of 50 µA cm^−2^ [58]. The diffusion coefficient of Li^+^ ions (*D*_Li_) in LCO thin films appears as a two-step behavior with a decrease of almost two orders of magnitude around 3.9 V (Figure 12). Due to the higher crystallinity, *D*_Li_ increases with annealing temperature (*T*_a_) and indicates a stable layered structure for *T*_a_ = 700 °C.

### 5.3. Solid-Electrolyte/Electrode Interface

Numerous studies have been devoted to the behavior of the solid-electrolyte/electrode interface (SEI layer). The sputtered LCO/LiPON interface was investigated step-by-step by photoelectron X-ray-induced spectroscopy. The SEI layer, ~10 Å thick, contains new nitrogen-containing species, i.e., NO_2_^−^ and NO_3_^−^ [98]. The reactivity of LCO thin films with a non-aqueous electrolyte, i.e., 1 mol L^−1^ LiClO_4_ in propylene carbonate (PC), has been investigated by several electroanalytical techniques, i.e., electrochemical impedance measurement, in situ Fourier transform infrared (FTIR) spectroscopy, and X-ray photoelectron spectroscopy (XPS). The formation of decomposition products, i.e., the organic surface layer, greatly depends on the crystal orientation and amount of the impurity (2 mol% Co_3_O_4_ cubic phase). The best electrochemical performance (229 mC cm^−2^ µm^−1^ in the voltage range 3.5–4.4 V) is attained for LCO films sputtered on Au substrate (*T*_s_ = 300 °C; Ar/O_2_ of 2:1; and Θ_dr_ = 8 nm min^−1^) with a (003) plane perpendicular to the substrate [99]. In the following experiments, the decomposition of the aprotic solvent mixture ethylene carbonate (EC) + diethyl carbonate (DEC) was studied by in situ FTIR spectroscopy. The electrochemical oxidations of EC:DEC occurred on charge even at 3.8 V vs. Li^+^/Li [100]. Finally, the same group reported that atomic force microscopy (AFM) images showed the decomposed products appeared during charge on the LCO film surface, which disappeared from the surface upon discharge at the potential lower than 3.9 V vs. Li^+^/Li [101]. The electrolyte solution containing lithium bis(oxalate)borate (LiBOB) showed that the absorption of BOB anions occurs at the LCO film surface above 4 V, preventing the decomposition of PF_6_^−^ anions of the Li salt [102]. A dense LiCoO_2_ microcrystalline buffer layer (−20 nm thick) was deposited by rf-sputtering between the cathode (LiNi_0.5_Co_0.2_Mn_0.3_O_2_) and solid electrolyte (Li_7_Al_0.1_La_3_Zr_2_O_12_ pellet) in an all-solid-state lithium battery [103]. Due to the large interfacial specific surface area and the excellent interfacial stability of the LCO thin film, the interfacial energy barrier was only 97 meV and the interfacial contact resistance was reduced by 1279 Ω.

### 5.4. Effect of Doping

**Figure 13:**
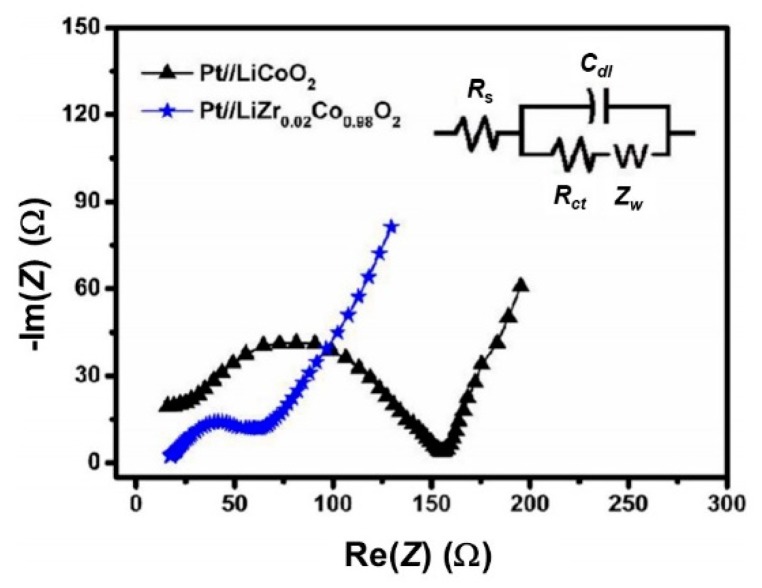
Nyquist plots of Pt//LiCoO_2_ and Pt//LiCo_0.98_Zr_0.02_O_2_ thin film cathodes. Inset displays the equivalent circuit model. Reproduced with permission from [106]. Copyright 2018 Elsevier.

Due to the rhombohedral/monoclinic phase transition in Li_x_CoO_2_, irreversible changes occur upon the charge process at *x* ≈ 0.5, which limits the specific capacity to 140 mAh g^−1^ or 69 µAh cm^−2^ µm^−1^. Reportedly, one of the significant approaches to overcome this problem is lattice doping by either isovalent or aliovalent ions that results in the stable cycling at high voltages (4.5 V) [104,105]. The influence of Zr doping on microstructural and electrochemical performance was investigated on a series of RF magnetron sputtered LiZr_x_Co_1−x_O_2_ thin films deposited on Au/Ti/SiO_2_/Si (100) substrates [106]. LCO films were deposited at the rate 13 nm min^−1^ on a substrate held at *T*_s_ = 250 °C under Ar/O_2_ (9:1) mix gas at working pressure of 0.6 Pa using a RF power of 130 W. 1.2-µm thick LCO films exhibit a (108) preferential orientation. A slight increase in lattice parameters and c/a ratio (5.01 vs. 4.98 for pristine film) has been noticed for the LiCo_0.98_Zr_0.02_O_2_ films, confirming the introduction of Zr^4+^ ions (0.72 Å) in the host lattice. It is assumed that the presence of some Co^2+^ ions is due to a charge compensation effect. Electrochemical properties of doped-LCO films were investigated by CV and GCD. The narrow anodic/cathodic peak separation in cyclic voltammograms and the high Li^+^ ion diffusion coefficient (1.8 × 10^−11^ cm^2^ s^−1^) indicate an enhancement of kinetics of Li^+^ ions by Zr doping. The Li//LiCo_0.98_Zr_0.02_O_2_ cell with non-aqueous electrolyte exhibited an initial discharge capacity of 65 μAh cm^−2^ μm^−1^ at a 1C rate with a fading of 3.8% after 80 cycles in the potential range 3–4.2 V. The extension to a higher voltage was not explored in this work. Improved kinetics is also evidenced by the Nyquist plots shown in Figure 13. One observes a decrease of the charge-transfer resistance (*R*_ct_) from 156 Ω (LiCoO_2_) to 60 Ω (LiCo_0.98_Zr_0.02_O_2_).

Ti-doped LCO thin films were prepared using a sputter Li-enriched LiCoO_2_ mosaic target including Ti metal strips [107]. The chronoamperometry measurements revealed an enhanced capacity of 69 μAh cm^−2^ μm^−1^ (245 mC cm^−2^ μm^−1^) at a 1C rate that retained to ~54 μAh cm^−2^ μm^−1^ at a 4C rate for LiCo_0.98_Ti_0.02_O_2_ thin film cathodes. The larger ionic radius of Ti^4+^ (0.605 Å) than that of the Co^3+^ ion (0.545 Å; in CN = 6) provokes a slight elementary volume expansion of 0.6% and produces a pillaring effect that result in an enhancement of the Li^+^ ion diffusion. Another reason for such a good electrochemical performance with aliovalent doping is based on the increasing Co^3+^ ions concentration and the lower concentration of Jahn-Teller Co^4+^ ions that cause spontaneous deformation and disrupt the LCO lattice on the charge process [108]. For a high doping, *y*(Ti) > 0.02, the ion exchange provokes more cation disordering and the appearance of Co_3_O_4_ spinel impurities [109]. When prepared at *T*_s_ = 250 °C using a 10% Li-enriched target, the Ti-doped LCO films exhibited a preponderant (104) orientation [110].

**Figure 14:**
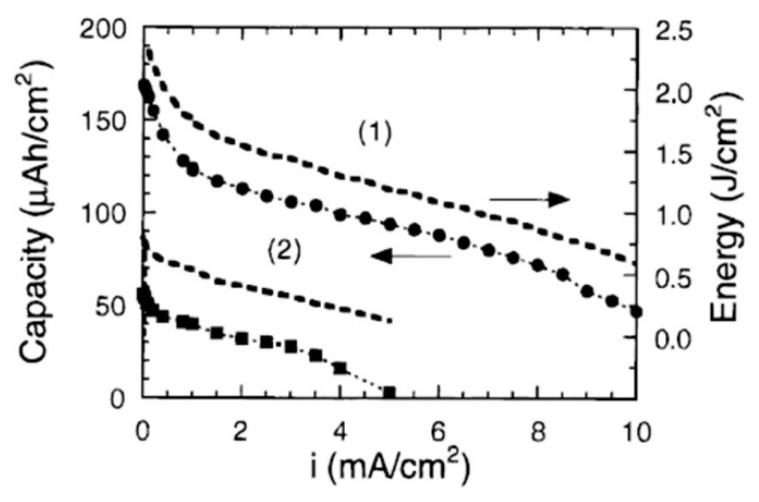
Variation of the discharge capacity (data points) and energy (dashed lines) against current density for LCO thin-film electrodes with preferential orientation. (1) 4-µm thick film with (101) and (104)-oriented grains in a ratio 84:16. (2) 1-µm thick film with 100% (003)-oriented grains. Reproduced with permission from [46]. Copyright 2000 The Electrochemical Society.

As a summary figure, the experimental results by Bates et al. [46] have clearly correlated the preferential growth of crystalline LCO films with sputtering conditions and resultant electrochemical properties. Figure 14 shows the variation of the discharge capacity (data points) and energy (dashed lines) against the current density for LCO thin-film electrodes with preferential orientation. Films were deposited on a Si/Co/Pt multilayer substrate in an Ar + O_2_ gas mixture in a ratio of 3:1 at a total flow of 20 sccm and a partial pressure of 2.7 pa. As an experimental fact, LCO films over 1-µm thick deposited at *T*_s_ ≤ 50 °C at a rate of 2 nm min^−1^ are 100% (003)-oriented grains, which demonstrates the lowest surface energy of the (003) plane. In contrast, 4-µm thick LCO films deposited at *T*_s_ = 70 °C at a rate of 1.3 nm min^−1^ exhibit 84% (101)- and 16% (104)-oriented grains (0% (003)); such a predominant texture was attributed to the large bulky strain energy in thick film [46]. As shown in Figure 14, the electrochemical features of thin electrode with (003)-oriented grains are greatly altered compared with the thicker LCO film. Of all these experiments, it appeared that the electrochemical performance of sputtered LCO films is a complex function of deposition conditions as demonstrated in this review paper.

## 6. Concluding Remarks

In this paper, we reported the successful use of the rf-magnetron sputtering technique for the synthesis of LiCoO_2_ thin films with adequate texture and microstructure that allows it to be associated with a solid-state electrolyte thin films applied in rechargeable lithium microbatteries. For rf-sputtered LiCoO_2_ films, experimental results have shown the strong influence of the growth conditions on the preferential orientation, microstructure, and stoichiometry, which govern the electrochemical performance of cathode films in microbatteries. The (104) orientation facilitates larger Li-ion transport at the electrolyte/electrode interface and an increase in the capacity than that of the (003)-oriented thin film. Such preferred texture has been obtained using the annealing process as low as 500 °C, which is a favorable condition for the device fabrication. Based on the optimization of parameters, good quality LiCoO_2_ films, from which more than half lithium ions can be extracted, delivered a specific capacity higher than 70 µAh cm^−2^ µm^−1^.

We focused attention on planar batteries. With this geometry, the areal energy density is limited. The thickness of the films is also limited to obtain good kinetics. 3D microbatteries are a promising design since it allows a scaling of the capacity by increasing the surface area of the 3D substrate, independent of film thickness. However, 3D TFBs have proven technologically very challenging to realize. The promise of a high capacity and stable microbattery has yet to be realized [111]. Nevertheless, efforts have already been made to model 3D thin film batteries for LiCoO_2_/graphite materials taking into account the issues with inhomogeneous current distributions [112,113,114], inevitable with such complex geometries. Sputtering is one of the techniques used to construct such devices that are still at the research level [115,116].

To date, lithium microbattery technology including LiCoO_2_ thin film cathode is almost mature and in the final form for actual applications. This class of power sources that belongs to the all-solid-state battery family including a lithium ion conductor (LIC) without undergoing liquid electrolyte leakage has the advantage of well-formed solid-electrolyte/electrode interfaces. Generally, LICs are safe with no risk of fire or explosion, thermally stable, and exhibit a wider electrochemical window than that of liquid electrolytes. However, the current density is quite low due to the poor ionic conductivity of the solid electrolyte such as LiPON. Development of a better electrolyte with the Li transport number close to unity such as perovskite-type oxides ((Li,La)TiO_3_), garnet-type frameworks (Li_5_La_3_Ti_2_O_12_), or nitride-based glass ceramics are good candidates. Deposition of these materials by rf-sputtering should be the best technique in terms of film quality. Another prospective for the future is the development of thin films on flexible substrates for wearable electronics applications. The recent fabrication of a Li/LiBON/LCO microbattery demonstrates good electrochemical performance, i.e., capacity retention of 90% over 1000 cycles under heavy bending and twisting conditions.

## Figures and Tables

**Table 1:** All solid-state Li microbatteries with a LiCoO_2_ (LCO) cathode film fabricated by the RF-sputtering technique.

Electrochemical Chain	Specific Capacity (µAh cm^−2^ µm^−1^)	Cyclability @ Current Density	Ref.
Li/LiPON/6.2 µm LCO/Pt/Ti/glass	40	40@20 µA cm^−2^	[22]
Li/1.5 µm LiPON/Pt/3.7 µm LCO	67	50@200 µA cm^−2^	[23]
Li/LiPON/0.5 µm LCO/Pt	50	140@10 µA cm^−2^	[24]
Li/1.4 µm LiPON/0.45 µm LCO/Au	40	800@0.4C (10 µA cm^−2^)	[25]
Li/LiPON/NASICON/LCO/Pt	15	50@0.01C	[26]
Li/Li_2.64_PO_2.81_N_0.33_/LCO/Pt/mica	22	800@10C	[27]
Li/Li_3.09_BO_2.53_N_0.52_/LCO/Pt/mica	44.3	1000@1C	[28]
Li/LiPON/LCO/Au/Ti/SiO_2_/Si	58	30@4C	[29]
Li/LiPON/LCO/Pt/Ti/TiO_2_/Al_2_O_3_	60	500@5C	[30]

**Table 2:** The ionic conductivity of LiPON films prepared by RF-sputtering.

Composition	Target	N/P Ratio	Conductivity (S cm^−1^)	Ref.
Li_4.2_PO_2.8_N_0.46_	Li_3_PO_4_	0.46	3.3 × 10^−6^	[33]
Li_4.2_PO_2.8_N_1.2_	Li_3_PO_4_ +Li_3_N	1.2	4.1 × 10^−7^	[34]
Li_2.971_PO_1.875_N_1.25_	Li_3_PO_4_	1.2	1.67 × 10^−6^	[35]
Li_3.3_PO_2.1_N_1.4_	Li_3_PO_4_	1.4	1.6 × 10^−6^	[36]
Li_2.9_PO_2.9_N_0.5_	Li_3_PO_4_	0.5	1.4 × 10^−6^	[37]
Li_4.0_PO_3.9_N_0.4_	Li_3_PO_4_	0.4	1.75 × 10^−6^	[38]
Li_3.2_PO_3.0_N_1.0_	Li_3_PO_4_	1.0	3.0 × 10^−6^	[38]

**Table 3:** Experimental conditions for the preparation of LCO thin films deposited by the rf-sputtering technique.

Atmosphere ^a)^	Power (W)	Deposition Rate (nm s^−1^)	Substrate	Substrate Temperature (°C)	Structural/Electrochemical Properties ^c)^	Ref.
3:1/55/1.0	100	3.2	Si (100) wafer	25	Influence of the target history and deposition geometry	[51]
9:3/12/5	100		Si/SiO_2_/Ti/Pt	250	*T*_a_ = 700 °C, *Q*_d_ = 61 µAh cm^−2^ µm^−1^; *R*_c_ = 74% after 50 cycles	[63]
3:1/40/0.5	80	1.6	Si/Ti/MgO/Pt	10	*T*_a_ = 800 °C, *Q*_d_ = 70 µAh cm^−2^ µm^−1^ @ 5 µA cm^−2^; *R*_c_ = 30% over 40 cycles	[64]
96:4/50/0.5	2.75 ^b)^	~0.3	Al_2_O_3_/Ti/Au	~110	*T*_a_ = 800 °C, *Q*_d_ = 60 µAh cm^−2^ µm^−1^ @ C/10; *R*_c_ = 95% over 100 cycles	[47]
9:1/-/0.5	4.4 ^b)^		Si/Pt and Cu foil	200	*T*_a_ = 700 °C, *Q*_d_ = 52 µAh cm^−2^ µm^−1^ @ 50 µA	[61]
5:1/150/0.2	130	0.03	Al foil	65	*c*, *Q*_d_ = 46 µAh cm^−2^ µm^−1^ @ 5 µA cm^−2^; *R*_c_ = 78% over 100 cycles	[65]
9:1/-/0.5	150	0.1	Si/Al/Li_2_O	25	(101)-oriented; *Q*_d_ = 40 µAh cm^−2^ µm^−1^@20 µA cm^−2^; *R*_c_ = 78%@640 µA cm^−2^	[48]
4:1/150/0.27	130	0.05	Stainless steel	25	*Q*_d_ = 44 µAh cm^−2^ µm^−1^@10 µA cm^−2^; *R*_c_ = 66% after 30 cycles	[66]
3:1/53/2.2	500		Al foil	25	*T*_a_ = 500 °C, *Q*_d_ = 50 µAh cm^−2^ µm^−1^@10 µA cm^−2^; *R*_c_ = 80% after 800 cycles	[25]
1:0/-/2.0	100	8.3	Au	25	Kinetics of (104)-plane. *D*_Li_ ≈ 10^−10^–10^−12^ cm^2^ s^−1^	[67]
2:1/-/0.5	200		Pt wafer	55	*P*ower of 200 W, *Q*_d_ = 61 µAh cm^−2^ µm^−1^@20 µA cm^−2^	[68]
40:1/20/0.14	500	1	Quartz/Pt	300	Thickness dependence; *Q*_d_ = 72 µAh cm^−2^ µm^−1^@0.1 mA cm^−2^	[23]
3:1/12/2	100		Si/Pt	25–600	*T*_s_ = 250 °C*, T*_a_ = 600 °C, *Q*_d_ = 50 µAh cm^−2^ µm^−1^@10 µA cm^−2^	[70]
9:1/-/0.5	50	0.8	Sapphire/SiO_2_/Al	25	*T*_a_ = 500 °C, thermal conductivity 3.7 W m^−1^ K^−1^ for Li_0.6_CoO_2_	[71]
9:3/12/0.5	50	0.02	Si/SiO_2_/Pt	25	*T*_a_ = 800 °C, *Q*_d_ = 27 µAh cm^−2^ µm^−1^@50 µA cm^−2^ after 150 cycles	[72]

a) Composition of the Ar:O_2_ gas mixture/flow rate in standard cubic centimeter per minute (sccm)/chamber pressure in Pa; b) Specific sputtering power in W cm^−2^; c) *T*_a_ = optimum annealing temperature; *Q*_d_ = specific discharge capacity; *R*_c_ capacity retention.
